# “May all Be Shattered into God”: Mary Barnes and Her Journey through Madness in Kingsley Hall

**DOI:** 10.1007/s10912-018-9517-1

**Published:** 2018-06-02

**Authors:** Adrian Chapman

**Affiliations:** Medical Humanities Research Centre, University of Glasgow, Glasgow, G12 8QQ Scotland UK

**Keywords:** Mary Barnes, Kingsley Hall, R. D. Laing, Anti-psychiatry, Metanoia, Spirituality

## Abstract

Contributing to renewed scholarly interest in R. D. Laing and his circle, and in the radical therapeutic community of Kingsley Hall, London (1965-1970), this article offers the first article-length reading of Mary Barnes’ and Joseph Berke’s *Mary Barnes: Two Accounts of a Journey through Madness*. This text offers views of anti-psychiatry ‘on the ground’ that critique the 1960s utopianism of Laing’s championing of madness as a metanoic, quasi-psychedelic voyage. Barnes’ story, too, reveals tensions within the anti-psychiatric movement. Moving beyond existing criticism of the text, Barnes, it is argued here, emerges as far more than an exemplary patient, victim or anti-psychiatric puppet. Particular attention is paid in this reading of *Two Accounts* to the following: the ways in which the spiritually inclined Barnes and the psychoanalytic Berke differ in this dual narrative text; the ways in which each differs from Laing; the metaphor of the journey; and the setting of Barnes’ story in the often conflicted, experimental household of Kingsley Hall.

Mary Barnes’ and her therapist Joseph Berke’s *Mary Barnes: Two Accounts of a Journey through Madness* is a text that can enrich our understanding of radical 1960s psychiatry in the UK. *Two Accounts* tells the story—or rather *two* stories—of Mary Barnes (1923-2001) and the anti-psychiatric community of Kingsley Hall, London (1965-1970), where her story of madness and recovery is set. Anti-psychiatry in Britain is chiefly associated with R. D. Laing, the Scottish psychiatrist most renowned for *The Divided Self* (1960), which presented madness as an intelligible response to an untenable situation, and *The Politics of Experience* (1967), a later text that aroused great countercultural interest in the 1960s—not only in the UK but also in mainland Europe and the US—in which Laing embraced madness as a higher form of sanity. In the present age of ‘big pharma,’ with the Diagnostic and Statistical Manual of Mental Disorders (now in its fifth edition [APA 2013]) and the biomedical model of mental illness having come in for vigorous criticism (Double 2006; Moncrieff 2009; Davies 2013), there has been a rekindling of interest in anti-psychiatry (Chapman 2014a, 2014b, 2015, 2016; Murray 2014; Staub 2011; Wall 2013). But despite renewed attention to Kingsley Hall (Fowler 2001; Harris 2012; McGeachan 2014), there has been no sustained re-examination of *Two Accounts.* Two recent texts where we might expect to find reference to Barnes instead make no mention of her. The psychoanalyst Darian Leader’s 2012 *What is Madness?* (which widely surveys first-person accounts of madness), and the historian Barbara Taylor’s 2015 *The Last Asylum* (a personal, feminist, and scholarly engagement with the nature of asylum) give no attention to Barnes. Here I offer the first article-length critical engagement with *Two Accounts*, the major text to come out of Kingsley Hall.

First published in 1971, *Mary Barnes: Two Accounts of a Journey through Madness* (1991) by Barnes, a one-time English nurse, and her therapist, Joseph Berke, an American doctor who came to England excited by Laing’s work, was translated into twelve languages. In the late 1960s and 1970s Barnes attracted media interest (e.g. Doyle 1968, Gillie 1969, Tyrer 1969), and the English playwright David Edgar based a 1979 play, *Mary Barnes*, on the Barnes and Berke text. The play, an elegiac defense of 1960s counterculture, presents Barnes’ story very sympathetically. What it does not and cannot represent, though, are the implications of the dual narrative form of Barnes’ and Berke’s text.

*Two Accounts* offers views of anti-psychiatry ‘on the ground’ that can widen our perspective on radical, countercultural psychiatry in the UK. Crucially, the accounts, while congruent in some respects, also diverge, revealing differing stances towards Laing and fractures within the anti-psychiatry movement, as well the development of Barnes’ ability to differ in outlook from those around her. Barnes’ understanding of her madness is closer to Laing’s than Berke’s. Very much like Laing in *The Politics of Experience* and similarly to current understandings of mental illness as a sign of spiritual emergency/emergence (Farber 2012; Mottram 2014; Razzaque 2014), she understands severe emotional disturbance in spiritual terms. Berke’s understanding, contra Laing, is psychoanalytic. Both Barnes and Berke, however, frame Barnes’ experience in terms of a journey, a metaphor that echoes, but also differs from, Laing’s metaphor of the voyage. Barnes’ *journey*, we shall see, is far more prosaic than Laing’s quasi-Conradian *voyage*. As Showalter begins to indicate, Barnes’ narrative differs from Laing’s view of how a ‘natural,’ and implicitly male, madness proceeds (1987, 235-36). Here I detail how *Two Accounts* foregrounds murderous aggression, fear and regression and how Barnes’ painful, slow recovery occurs in the often-fractious environment of Kingsley Hall, a community that is ambivalent about her journey into madness and imposes limits on it. Berke’s care, too, is conflicted; under great pressure, he even strikes Barnes. We are far, then, from Laing’s vision in *The Politics of Experience* of harmonious treatment for psychotics undergoing healing inner voyages*.* There Laing looked forward to the melting of boundaries between doctor and patient. *Two Accounts* shows these roles to be still in play and renders problematic Laing’s 1960s utopianism. Yet Barnes’ condition, both she and Berke attest, improves, and Kingsley Hall certainly challenges contemporaneous mental health treatment. Barnes slowly learns to symbolize her rage and guilt through religious paintings and brief therapeutic narratives. The writing of *Two Accounts* is therapeutic for Barnes, too; and here we must be careful not to read the text as attesting to therapeutic failure due to divisions between the two narratives. Such divisions, rather, can be read as signs of therapeutic advancement. Certainly, the form of the text, rare in accounts of mental illness, is significant. The dual narrative form, which inevitably results not only in parallels but also in differences, lends itself to representing Barnes’ (and Berke’s) project of enabling her autonomy.

In this article I move beyond existing criticism of *Two Accounts* that reduces Barnes to Laing’s exemplary patient, little more than a victim or puppet (Szasz 1976; Showalter 1987) or that sees her as a victim and advocate of ‘oedipalising’ therapy (Guattari 1996). *Two Accounts*, I argue, rather reveals Barnes precariously carving out her own subjectivity. Particular attention is paid in this reading of *Two Accounts* to the following: the ways in which the spiritually inclined Barnes and the psychoanalytic Berke differ in this dual narrative text; the ways in which each differs from Laing; the metaphor of the journey; and the setting of Barnes’ story in the often conflicted, experimental household of Kingsley Hall.

## R. D. Laing’s ‘template’ of madness as a voyage

In *The Divided Self* (1960), Laing’s first book, his emphasis was on the intelligibility of madness. In *Sanity, Madness and the Family* (1964)*,* the individual’s symptoms are dissolved in the nexus of the family, which becomes the object of analysis. The model of madness presented in the 1967 *The Politics of Experience* differs again: it is countercultural and psychedelic. Here madness is a route to expanding experience, renewing spirituality and recovering authenticity. While the LSD guru Timothy Leary wrote of the need for the LSD user to have a ‘baby-sitter’ in order to facilitate the spiritual journey of a drug-induced ‘trip’ (Leary et al. 1964), Laing wrote of the mad person’s need for support to fully experience a psychotic voyage (a madness trip, we might say) in which a socially adjusted false self might melt away and a spiritually renewed true self emerge. Such support forms part of the setting in which psychotic experience might be therapeutic. In “Metanoia: Some Experiences at Kingsley Hall, London” (1972), Laing further aligns the voyage into madness with Leary’s conception of the LSD trip. He writes that the “biochemically induced … trip has its natural analogue in what I suggest be called a *metanoiac* voyage (from metanoia: change of mind)” (12). The change of mind that occurs depends (just as with the LSD experience) on the setting in which it takes place. Given the right setting, and Kingsley Hall was Laing’s attempt to construct precisely that, psychosis might be a means of radically expanding experience, a natural high offering an accelerated spiritual transformation. This view is satirized at length by Clancy Sigal, a one-time Kingsley Hall resident and individual patient of Laing, in his *Zone of the Interior*.

Laing prefers the term “voyage” to “trip.” His use of the voyage metaphor aggrandizes madness: there are, he writes, “many occasions in which to lose one’s way, for confusion, partial failure, even final shipwreck,” but just as we “respect the explorer, the climber, the space man,” so the schizophrenic, too, the explorer of inner space, is deserving of respect—in fact, worthy of even greater respect as far less is known about inner than outer space (Laing 1967, 104-105). Here Laing’s mad person, I would argue, is a psychic cosmonaut enacting a critique of both the capitalist West and the Socialist world and their space race that serves only to alienate humanity further from itself. Experience is indeed political. What is more, madness is presented as offering the dimension of resistance absent from what the political philosopher Herbert Marcuse referred to as one-dimensional man, someone numbed by the advances of technocratic and consumer-orientated civilization (1964).

Madness, too, for Laing can be an adventure. This is brought out in *The Politics of Experience* in his presentation of the ten-day voyage of sculptor and friend, Jesse Watkins. Laing emphasizes the Conradian, almost *Boys’ Own*, story of Watkins’ years as a merchant seaman and sailor: “During his career at sea he encountered shipwreck, mutiny, murder.” Watkins’ breakdown is sudden, dramatic, beginning with him experiencing time going backwards. He becomes convinced he is dead. In his mind, he returns to babyhood and to a form of animal life. His reading of newspaper articles sets off long chains of association having, apparently, extraordinary significance. He experiences the Stations of the Cross and the presence of God. After a brief and basically supportive period of hospitalization, he returns to the world 10 days later with sharpened senses and the impression of having undergone something fundamental and necessary. “Can we not see,” adds Laing “that *this voyage is not what we need to be cured of, but that it is itself a natural way of healing our own appalling state of alienation called normality*?” [italics in the original] (1967, 120, 126). For Laing, in high 1960s countercultural mode here, the mad voyage potentially enables the recovery of the full, authentic self.

Watkins says that those undergoing a mad voyage need a “sheet anchor,” an emergency support, someone to stop them going utterly adrift. Laing remarks that such a person, who might also function as a guide to the process of dis- and re-integration, could be someone who has already undergone a successful mad voyage. “Psychiatrically,” Laing says, “this might appear as ex-patients helping future patients to go mad.” He tells us that “Among priests and physicians there should be those who are guides, who can educt the person from this world and induct him into the other. To guide him in it: and to lead him back again” (1967, 106). Laing’s one-time colleague David Cooper writes similarly about the figure of the witness, someone qualified through personal experience of madness to support another through a journey of discovery (1971, 31-35). Laing’s and Cooper’s view that the treatment of madness ought properly to be de-professionalized anticipates mental health service users’ and survivors’ promotion of peer support as an alternative to institutional care.

For Laing and his anti-psychiatric colleagues, the panoply of ordinary treatments for psychosis is likely to arrest the natural process of madness: “We are so busy ‘treating’ the patient, whether by chemotherapy, shock therapy, *milieu* therapy, group therapy, family therapy, psychotherapy,” claims Laing, that there is little chance of successful rebirth through madness (1967, 102). At a stroke, he dismisses not only biologically based models of madness and drug treatments but also the hospital-based therapeutic community movement identified with Maxwell Jones in the UK, as well as psychotherapy and psychoanalysis.

In *The Politics of Experience* chapters on schizophrenic experience and madness as a voyage, psychoanalytic terminology is employed sparingly—and sneeringly. Laing clearly wants to distance himself from psychoanalysis. The word “regression,” for instance, appears only once and then as an example of how madness tends to be misunderstood. Laing’s only use of “regressed” is again to indicate that the very word represents misunderstanding. In his view, psychoanalytic discourse fails to recognize that the psychotic “may be irradiated by light from other worlds,” and this zone of experience cannot be reduced to “a battlefield wherein psychological forces, derived or diverted, displaced or sublimated from their original object cathexes are engaged in an illusionary flight.” Psychoanalysis, in Laing’s view, is incapable of coming to terms with the transcendental nature of madness. The mystical, for Laing and, we shall see, for Barnes ought to be taken seriously. Indeed, the schizophrenic might well be “a hierophant of the sacred” (1967, 92, 137, 114, 109). Employing a phrase that recalls Aldous Huxley’s *The Perennial Philosophy*, Laing associates the experience of psychosis with the “well-springs of all religions.” For Huxley, “the metaphysic that recognizes a divine Reality substantial to the world of things and lives and minds”—the perennial philosophy—is present in all religions, from those of what he terms “primitive peoples” to the beliefs of “higher religions” (1947, 1). Laing’s schizophrenic, then, is not merely spiritually well-attuned; the experience of madness rather makes one representative of the truth at the heart of all religions. Such truth, Laing claims, lies buried by “*egoic* consciousness,” itself nothing more than “a preliminary illusion, a veil, a film of maya.” Madness, however, might be a route to genuine sanity in which “the ego now being a servant of the divine, [is] no longer its betrayer” (1967, 109, 112-113, 119). Laing’s view of the need to transcend the socially adaptive, and mendacious, ego in order to attain a higher self relates him closely to Paul Heelas’ view of the key features of Self-spirituality. Heelas maintains, too, that Self-spirituality characteristically offers means of attaining the higher self state (1996, 18), and the proto-New Age Laing presents his readers with a specific technology here: the transformative psychotic voyage.

The term Laing employs (not in *The Politics of Experience* but in his 1972 essay) to present “transformation of a potentially liberatory kind” is “metanoia”: “It is a traditional term,” he says. “It is the Greek New Testament term, translated in English as *repentance,* in French as *conversion*. Literally, it means: a change of mind” (16). Although he does not refer to Carl Jung, Laing probably picked up the term from the Swiss psychiatrist for whom metanoia denotes “a mental transformation” that often marks the second part of one’s life (1977, 26). Laing’s choice of term sanctifies his conception of madness. While he does not to link this view to others’ work, the view that madness may be a form of suffering that leads to spiritual advancement can also be found in contemporaneous work of Julian Silverman (1967), Joseph Campbell (1972), and John Weir Perry (1974) as well as in the work of the author Doris Lessing (1962, 1969, 1971). Keying into what we might call a wider metanoic ‘mood,’ then, Laing issues a call for a return to an ancient but now regrettably little valued form of spiritual development that involves a perilous but ultimately rewarding conversion experience.

While Laing does not unreservedly advocate the mad voyage and emphasizes its potential perils, he certainly presents the voyager as courageous: a daring and, as Showalter points out in *The Female Malady*, implicitly male pioneer of psychic exploration; someone, by implication, who critiques the priorities of both the First and Second Worlds. Laing further aggrandizes madness by relating it to the experiences of “artists who have become shipwrecked” during an unsupported journey, and he refers to the examples of “Hölderlin, John Clare, Rimbaud, Van Gogh, Nietzsche, Antonin Artaud” (1967, 116). In characterizing the mad person as having privileged access to creativity, Laing rehearses a commonly made link between insanity and artistic production, a connection also discussed by Gilman (1985, 575-597).

Imbued with creativity, a path to authenticity and fullness, a means of transcending quotidian illusion and moving towards enlightenment, madness (for Laing) is clearly a very privileged experience. It is not surprising, then, that Laing came to be seen as a prophet of the liberatory potential of madness. This liberatory potential, and in particular its spiritual element, is picked up upon by Seth Farber in *The Spiritual Gift of Madness* (2012). For Farber, in contradistinction to the far more conservative views of most who now understand madness as a possible sign of spiritual emergency/emergence (e.g. Mottram 2014; Razzaque 2014), madness is unproblematically redemptive. While Farber goes further than Laing, going so far, for instance, as to claim that the schizophrenic can be the catalyst of messianic revolution, he certainly registers Laing’s zeal—proselytizing, even—for the redemptive nature of madness. And spiritual redemption, we shall see, is a key part of Barnes’ story.

## *Two Accounts*: critique and development of Laing’s ‘template’

While it is not difficult to find first-person accounts of recovery that are introduced by a doctor or therapist and bookended by a professional’s afterword, and while accounts in which the flow of narration is interrupted by a spouse or family member are again not terribly unusual, there are few texts that interlace accounts by both someone in therapy and his or her therapist.^1^ Perhaps the best well-known example of the split narrative form is *Every Day Gets a Little Closer* by Irvin Yalom and his pseudonymous patient Ginny Elkin (1974). Here the text’s two narratives are densely interlaced with very brief sections by patient and therapist following each other rapidly, and, like some epistolary novels, writing is part of the story: Yalom and his patient agree to write accounts of each session and exchange them, and Elkin’s writing is *in lieu* of payment. *Two Accounts* is very different. Barnes’ and Berke’s writing is all done after the therapy. What is more, while both Barnes and Berke write three sections each of the text’s six parts, Barnes’ sections are far longer. Unlike *Every Day Gets a Little Closer*, the disturbed person’s name comes first on the book’s jacket and spine; and, rather than writing pseudonymously, Barnes writes in her own name. Indeed, she *must* write in her own name: the text’s purpose is to present her story of self-realization. In Yalom’s text, sections are headed by “Dr Yalom” and “Ginny”; in *Two Accounts*, we have just “Mary Barnes” and “Joseph Berke.” Clearly, readers are urged to accept the text as primarily Barnes’ story and principally the text of Barnes, someone given the same, or even superior, ‘billing’ as her professionally qualified carer.

The first edition of the text ends with Berke’s chapter, “Untangling Mary’s Knot.” The title appears to present Berke as the masterful professional who has successfully resolved Barnes’ difficulties. But this is a very brief chapter mostly concerned with the closure of Kingsley Hall, Barnes’ exhibition and how his relationship with her has changed. While Berke does present his psychoanalytic understanding of regression and remarks on how this differs from Barnes’ spiritual understanding, a detailed exposition of “untangling” promised in the chapter title is absent. The chapter certainly does not subordinate Barnes’ material. In later editions, we are given epilogues by Barnes and Berke, and the power of the “Untangling” chapter is reduced further by the authors’ fond remembrance of one another and Kingsley Hall, and, in Barnes’ epilogue for the 2002 Other Press edition, by the valedictory tone of an elderly woman close to death.

For Showalter, the differences between the two accounts are problematic. Both she and Guattari (1996, 53-54) note that as a girl and young adult, Barnes had desired vocations (writer, journalist, painter, doctor) commonly regarded as befitting only men. Certainly, Gauttari is right in implying, as Showalter says explicitly, that Berke fails to recognize the gendered nature of Barnes’ madness and the ways in which her madness is constituted by social forces beyond the family. As Showalter puts it, “What Berke sees as penis envy” can certainly be read as “envy of male mobility, status and independence” (1987, 235). For Guattari, Barnes does not achieve autonomy; far from it, the patient becomes the analyst par excellence, the evangelist of both anti-psychiatry and the family, who has succeeded in “magically denying social reality, and avoiding all connections with real fluxes.” And her art exhibition proves her only to be “like the star of a variety show” (1996, 49, 54). Szasz presents her as a victim, albeit a willing one, of anti-psychiatry (1976). If Guattari’s particular target is the family and psychoanalysis’ promotion of it, Showalter’s animus is reserved in particular for Laing, who epitomizes male psychiatric power.^2^ For both Showalter and Guattari, Barnes’ journey is not a success story. She certainly does become an anti-psychiatric celebrity, for which Guattari and Showalter deride her from the Left, as does Thomas Szasz from the libertarian Right. He presents her as a dupe who has done the bidding of countercultural conmen whose con is so powerful that they think of themselves as healers rather than psychiatric snake oil salesmen. Yet she authorizes her own story, and, as we shall see, it is her *own* story, one that clearly differs from that of her therapist. As Daniel Burston remarks, in the writing of *Two Accounts*, “no effort was made to privilege one narrative over the other as to what really happened between therapist and patient—surely a first in the clinical literature” (1996, 83). The differences in the accounts can be read as representing the successful development of Barnes’ autonomy. Not only does Barnes authorize her own account but she also tells us that she recovers. Are we simply to dismiss her words? To do so, and to view her as dupe or victim or perhaps a ‘bad feminist’ as some women writers of madness narratives have been viewed,^3^ would be to deny her agency and to silence her.

The first sign of Barnes’ and Berke’s kinship with, and difference from, Laing’s version of madness as a voyage is the word “journey” in Barnes’ and Berke’s title: *Two Accounts of a* Journey *through Madness.* The word suggests a passage, a crossing or expedition but lower in register as rather less grand than “voyage,” Laing’s preferred metaphor. This difference signals that *Two Account*s is to be a more prosaic account of madness. Nevertheless, Barnes’ journey is one that takes her through the extreme experience of madness, and “journey” is a word associated with learning (Turner 1998). The title raises the question of quite what Barnes and Berke have learnt and what readers might learn from their two accounts of madness. It is instructive to remember that prior to life at Kingsley Hall, Barnes had been a teacher of nursing and Berke an early member of the counter-cultural Free University of New York (Berke 1969, 212-22). *Two Accounts* is an attempt to educate and persuade us of something about Mary Barnes’s journey and about Kingsley Hall. To this end, both writers draw on the authority of first-hand experience. We shall see, though, that they do not teach quite the same lesson.

The journey metaphor provides coherence to the text. After recounting her life prior to Kingsley Hall in Part One, which serves as a prologue, Barnes’ journey begins. She departs from the world, setting aside her job and the struggle to continue functioning as a ‘normal’ person and moves into Kingsley Hall in 1965, aged forty-two. The text closes with a focus on an exhibition of Barnes’ paintings at Camden Arts Centre in 1969: the journey is over, and she can return to the world, having ‘found’ her ‘true’ self—indeed, having exhibited it. The exhibition is presented by Barnes as an evening of joy and recognition, with her brother and cousin present as well as friends, acquaintances and people from her life as a student and nurse. And Berke was there: “The big bear who had caused all the painting” (1991, 329). Rhetorically, ending with the exhibition appears to act as a means of narrative closure—a tying up of loose ends, a ‘natural’ ‘full-stop,’ *and* a proof of the efficacy of the radical therapy Barnes underwent at Kingsley Hall. Having been supported by Berke in her journey—how unlike the unsupported artists mentioned by Laing—her madness becomes a source of creativity, and she ‘arrives’ at her vocation as an artist. What is more, Barnes’ ‘true’ self is by the close of the text one that can fruitfully differ from Berke and from those around her. It is this difference that is key and here that the true closure of the text lies: not so much in the successful exhibition that ends the text but rather in Barnes writing an account of her life at Kingsley Hall and, in particular, the differences between her account and that of her therapist that imply the development of her successful ego differentiation.

Barnes begins by telling the story—authoring her own case history, we might say—of her life prior to Kingsley Hall. Her account of her early life and the development of her madness is filtered through the lens of anti-psychiatric understanding. She says her family “was abnormally nice”; voices were never raised: “The air was cold yet a storm was always brewing.” This unsettled and unsettling niceness associates her family with those of the schizophrenics presented in Laing and Esterson’s *Sanity, Madness and the Family* (1964) and Cooper’s *Psychiatry and Anti-Psychiatry* (1967)*.* These texts are echoed again in the presentation of Barnes’ brother Peter who as a young man is hospitalized as a schizophrenic and, it is implied, served as a container for the wider disturbance of the family nexus. He is portrayed as someone who attempted an escape from an untenable situation and whose deviance had to be marked with the sign of schizophrenia. “Such was the fear of truth,” we are told. Like Laing (and David Cooper), Barnes associates madness and truth: “Madness was a step on the way to truth. It was the only way.” Madness, too, in a very Laingian gesture that recalls *The Divided Self*, is linked by Barnes to freedom: “Peter was instinctively seeking freedom” (1991, 14, 17).

Far from asserting, however unconventionally, her own desire for freedom, Barnes experienced herself as being peculiarly absent throughout her childhood. She writes, “I would seem to go away, right away from everything and everywhere. I didn’t belong anywhere … There was a feeling of deadness, of being in a blind alley. My soul was musty, like a cobweb in the dust” (1991, 26). What is more, she literally had no voice for a time. She was a late talker, to the extent that she was taken to the doctor. *Two Accounts* is concerned with her finding words and images, and this search is very much part of her journey. While her brother broke down, she tells us, she went on to find solace in Catholicism and to train as a nurse, becoming first a district nurse, then entering the army and nursing in Egypt and Palestine, and finally training in England as a nurse educator. Then she broke down and was hospitalized as a schizophrenic in 1953 “for one year in Saint Bernard’s Mental Hospital” in West London where she was “put in a padded cell and was tube fed,” silenced, in other words. Despite then living in a convent and in supported housing, she felt the need for further support. She found her way to Laing through a contact from nursing education. “You need analysis twenty-four hours out of twenty-four,” he told her. “In one year’s time I will have a place and there you can stay and have treatment” (1991, 26, 13, 69).

It can be difficult at times when reading Barnes’ narrative to discern basic facts, such as dates and locations. This tendency, I suggest, might be related to Barnes’ difficulty in experiencing herself as fully separate from others. It is as though the reader and Barnes are ‘of the same mind,’ and there is no need to expand on details the reader will already know. She was cared for at a convent in Herefordshire but no name is given and quite how long she was there during her first breakdown is unclear. Sometimes names of people are given, but identities are unexplained. The text, of course, has been edited, and we have no way of knowing what revisions were requested or made. In the unpublished and presumably unedited (or at least unprofessionally edited) sequel to *Two Accounts*, though, Barnes’ tendency to name people as though the reader knows perfectly well to whom she is referring is even more marked.^4^ Barnes identifies (as does Berke) the lack of differentiation of herself from others as a significant problem and reason for her breaking down. Being madly bound up with other people and even mere things, it seems to Barnes that she is involved in everything and everything is involved in her. Shopping with Berke, for instance, she is alarmed that he buys fish and not duck. Thinking that the Kingsley Hall community would be dining on duck, Barnes thinks that it is her fault Berke is purchasing fish, but the household has simply decided to have fish. Again, when the bathroom heater breaks down, Berke has to assure Barnes that it is nothing to do with her (1991, 123). Barnes’ challenge—the chief test of her journey—is to find ways of accounting for herself in which she is not accountable for everything.

A significant reason for Barnes’ being ‘con-fused’ with others, it seems, is her difficulty in letting others be. It is particularly difficult for her to accept that Berke has his own life to lead. Chapter 8 is subtitled “*The pest – IT, my anger.”* “It” explodes when Barnes refuses to allow Berke to leave the Hall to attend to patients at the Langham Clinic on the other side of London. Berke, desperate to retain his independence in the face of Barnes’ ongoing attempts to control him, explodes too, hitting Barnes across the face and then leaving. Accentuating the drama through the use of the historical present, Barnes writes that the “blood pours down my white blouse on to the floor. A big red splash.” She is proud of the “big red splash” and wants to wear her bloody blouse to dinner. Another time, again oppressed by “It”—“IT, my anger, seemed to split and tear at me, cold and frightened and fragile,” she writes— she runs out of Kingsley Hall, declaring that she is going to admit herself to a mental hospital. Again, Berke strikes her, saying, “Oh why do you make me *do* this?” And here it seems that Berke, horrified at his own behaviour, is bound up with Barnes. Again, Mary’s nose bleeds. The two embrace, and Barnes cries deeply with great relief. “I never loved Joe so much,” she says. “Quite hard, horrible and hating, Joe brought me back. The big bear, with a flop of his paw, had saved me” (1991, 131-35). Barnes presents herself as having been a naughty child relieved to be reconciled with her father. Her images are sexually evocative. We are told of weeping and loving embraces, and it is as though Berke has penetrated Barnes, taking her virginity and causing blood to flow. In David Edgar’s play *Mary Barnes*, Barnes and Eddie (the Berke stand in) roll around playing at being crocodiles. The play offers the possibility of foregrounding a parent-child, and sexualized, element in the therapeutic relationship that is already present in *Two Accounts.*

Berke’s adoption of a paternal role, feeding Barnes milk, bathing her and putting her to bed, his violence and the sexualized relationship between him and Barnes place him a long way from the traditional role of doctor, and a long way, too, from the facilitative non-medical guide envisaged by Laing. Reflecting with difficulty, he says, on his own violence, Berke writes that he “was horrified, and thought, ‘What way is this for a doctor to treat his patient’?” Formally, there were no doctors and patients at Kingsley Hall, only residents. However, as he acknowledges, “those who had been trained as doctors found it difficult not to relate to others as doctors. Similarly those who had been trained as patients found it difficult not to relate to others as patient.” Mary, above all, he tells us, found these roles difficult to overcome. Nevertheless, it was equally challenging for visitors to determine who were the patients: “Nine times out of ten their observations about who was who were dead wrong.” Berke reflects upon his “mental double take” about being a doctor, and explains that it “was an expression both of the anxiety at seeing her bleed, and of guilt about transgressing a role which I had not consciously been playing, but with which, unconsciously, I was obviously still involved” (1991, 245-46). Berke’s words cast a negative light on Kingsley Hall’s founders’ optimistic 1960s ideal of melting boundaries and role divisions. Such a view is expressed by Leon Redler, a key member of the Hall, who, recalling the establishment of the household, wrote that it was hoped that the household would provide a place where people could “inquire into the possibilities of coming to know and be ourselves, with each other, as men and women, all essentially, in the same boat” (MS Laing L 221/15A). Kingsley Hall was not an ordinary mental hospital but a countercultural venture. Archival sources record a remarkable fluidity with which residents moved between the roles of professional, resident and intellectual.^5^ However, the patient-doctor roles were still operative. While not evident to visitors and flatly denied by sympathetic Kingsley Hall commentators (e.g. Schatzman 1972; Gordon 2010, 5-27), this becomes evident to Berke, to whom it recurs in a moment of ironic reflection that undermines the image of the Hall as a utopian experiment. The values and practices of the medical world beyond the Hall could not be entirely kept outside.

Barnes, who at first cannot bear anyone else having access to Berke, gets better, but her recovery is not linear, and it is far from ‘textbook’ Laingian. Laing’s aggrandizing term (absent from *Two Accounts*) for the return to the world after a period of radical withdrawal is “neo-genesis.” This can be, he tells us, “an oscillating movement: the graph can go up and down: it need not be a smooth parabola back and forward.” Nevertheless, he writes, in his only published written commentary on Barnes, “This woman [Barnes] came back over a period of five to six weeks” (1972, 18). Barnes’ own account, however, presents far more ‘oscillatory’ recovery (descending into states of withdrawal again four times, for instance, in 1967). But she begins painting and writing and is able to start interacting with other residents—thus finding ways through words and images of symbolizing her experience. Her coming ‘up’ is signaled by her being able to dedicate time to Catherine, a severely disturbed resident of Kingsley Hall. Barnes eventually becomes one of the former patients aiming at facilitating madness to whom Laing refers in *The Politics of Experience*. More than that, she becomes an evangelist of madness, wanting her brother to undergo an experience similar to her own. She becomes the explicit spokesperson for what is strongly implied but never clearly said in *Politics*: Go mad; it will do you the world of good, refreshing your spirit and returning you to your true self. It is left to Berke to point out that Peter’s path might be different to hers, and this is something Barnes comes to realize.

It is Berke who suggests that Barnes paint. Her first paintings, done on wallpaper, are accompanied by brief stories or little anti-psychiatric fairy tales. In *The Mermaid Story*, for instance, a mermaid stranded on dry land, literally out of her element, finds a way back into the sea; in *The Wind and the Flowers,* flowers are uprooted by wind to an uncongenial environment, but their seeds are carried to a place where they can flourish. *The Egg and the Sea* is almost the mirror image of *The Mermaid Story*: a woman finds herself ill-equipped for life in the sea and eventually finds her way on to land where she can live at ease. These are all stories about moving—journeying—from one environment into another where organisms might flourish. *The Death of a Family* is a tableau in which a boy and his father and mother all die from their failure to live with and express fear, and is a story that relates to Barnes’ own family’s failure to symbolize disturbing emotions (Barnes and Berke 1991, 139-41). It is important to note that while writing is clearly therapeutic for Barnes, she is not involved in anything like a program of writing therapy. There was no formal program of therapy at all in Kingsley Hall. In the fairy tales, though, we can note a foreshadowing of the therapeutic significance of Barnes’ writing of her *Two Accounts* narrative, written later.

Of more importance to Barnes than her writing, and of more interest to others, were her paintings. The Harcourt Brace 1971 edition, reprinted by The Other Press in 2002, gives far more prominence to artwork than other editions. Black and white images show Barnes finding ways of representing her rage through art. Two pictures, for instance, show her with her mother: a small and vulnerable presence towered over by a persecutory maternal presence. The words offered in explanation of one image are, “My mother and me with a sore throat and how I feel ill all over. To tell my mother”; for the other image the words are simply, “Me hating my mother.” We are also given a playful image showing Barnes and “a crocodile eating Joe—eating, crunching, gobbling, biting,” which suggests Barnes reflecting, through art-making, on her child-like games with Berke. The color plates, of large, expressionistic paintings on canvas or board are all religious in theme: *Peter Before Christ*, *Gathering Manna,* a triptych of Mary and Elizabeth, *The Blinding of Paul*, and *The Resurrection—*a subject that Mary, clearly working out the meanings of her own ‘rebirth’, painted several times (Barnes and Berke 2002, 98-99, 210-211). These paintings reveal Barnes’ spiritual concerns. It is to be hoped that if there is another edition of *Two Accounts*, we will be given even more images of Barnes’ artwork. Such images foreground the importance of symbolization beyond narrative, something that, as Angela Woods (2011) has pointed out, the field of medical humanities has tended to under-appreciate in its emphasis on storytelling.

Barnes writes that her “painting had emerged from black lines and breasts on the walls and paintings in shit, to moving figures and scribble on paper” (Barnes and Berke 1991, 152). She soon focused exclusively on hand painting in often-large artworks of swirling lines and bright colors, with almost exclusively religious themes. She is recognized as a fellow artist by Felix Topolski and Harry Trevor and by Jesse Watkins, the sculptor who had undergone Laing's ideal voyage of self-exploration. In the run-up to her Camden Arts Centre exhibition in North London, she became a counter-cultural celebrity of interest to the mainstream media. She is featured, for instance, in the 1968 BBC documentary *A True Madness* as well as in a *Vogue* article by Brian Inglis in 1969.

Her paintings were exhibited at the 1967 Dialectics Liberation Conference at the Roundhouse in North London, an event attended not only by Laing and Cooper as speakers but also Herbert Marcuse, Stokely Carmichael and Allen Ginsberg. Barnes, however, writes nothing about this event besides mentioning her little exhibition. As David Edgar points out in his 1991 introduction to *Two Accounts*, the Dialectics conference clearly related anti-psychiatry to wider political critique; but the concern with politics in *Two Accounts* is rather with the micro-politics of the household (i-xviii). The world beyond the Hall rather fades from view. Laing’s indictment of technocratic society in his *Politics* is eclipsed in *Two Accounts* by the day-to-day problems of living in an alternative community.

## Household politics

In *The Politics of Experience* Laing’s consideration of the supportive environment of the mad journey goes no further than the voyager-guide relationship, but in reading *Two Accounts* we must also consider the nature of the domestic physical and social environment, the household. Kingsley Hall in Bow, East London, had been established by two philanthropic sisters early in the twentieth century, and they ran community services from there. Inscribing the anti-psychiatric community in the history of resistance and radical struggle, Berke mentions that Gandhi stayed in the building in 1931 when attempting to negotiate Indian independence. However, the Hall had largely fallen into disuse by the 1960s and was offered at a nominal rent to the Philadelphia Association, the mental health charity set up by Laing and others in 1964. The place was spacious: a large meeting room, rooms that served as bedrooms, a games room, a dining room, and a kitchen, a roof garden and a self-contained flat on the roof (Barnes and Berke 1991, 225-8).

Having considered other places, Laing settled on Kingsley Hall as a place for a radical community. Laing spoke not of hospitals but rather “places of hospitality” that might be asylums in the etymological sense of the word: safe places (Evans 1976, 158). In his 1966 poem, ‘Prayer for a Place’, he envisages such an asylum as a place of pure being, somewhere “to dance and sing” and “to burst with agony and with ecstasy.” In this place, where one can “cry and scream” one might find “a place to live,” somewhere “to love / a place to be.” The image of the Hall as a “place to be” is undermined by Laing, however, in a discussion document for members of the Philadelphia Association that was appended to, and circulated with, the poem. In the attached document, Laing presents the Hall as chaotic, and, in a possible gesture of reconciliation aimed at Aaron Esterson, who desired greater structure in the Hall, Laing gestures tentatively towards a tighter from of organization (MS Laing L221/23). The utopian vision of Laing is again undermined by *Two Accounts.*

In Laing’s poem there is an implicit and very countercultural hierarchical opposition drawn between being and having, an opposition that Eric Fromm would go on to make explicit in his 1976 *To Have or to Be*. But we learn from *Two Accounts* that the issue of ownership and who ‘had’ control of space played a significant part in the life of Kingsley Hall. Barnes admits that at least at the beginning of the community, “In my mind it was me who controlled Kingsley Hall.” Just as *Two Accounts* is divided between two authors, so Kingsley Hall became split around two figures and two visions: Aaron Esterson wanted a more structured community with clear-cut rules and a medical director; Laing was content for the community to muddle along in a less structured fashion. Berke relates how it was at one time impossible to invite anyone into the community because agreement beforehand had to be gained from both camps (1991, 101, 253-5)—and here Berke highlights a fracture in the household that has been commented upon by other residents (see Cobb MS Laing L169/1-3; Zeal 2006).

Barnes herself is the cause of much consternation within Kingsley Hall. Her preoccupation with her feces—playing and painting with them—is a prominent feature of the text, and, as Ann Scott notes, in reading *Two Accounts*, we come up against society’s taboo against interest in feces (Barnes and Scott 1989, xxvii). Alison Torn (2012) reads Barnes’ fecal preoccupation in Bakhtinian terms as a carnivalesque expression of the orifice- and bodily product-precoccupied grotesque body. While clearly Barnes delights in her fecal matter, it is important to note that she is not given license to follow her ‘journey’ *any* way she wanted. Everyday values were not subject simply to carnivalesque inversion. Her taking up painting rather than smearing her feces on walls solved a smelly and disturbing problem for residents that might otherwise have led eventually to her expulsion from the Hall. Barnes’ desire to be tube-fed in order that she might return to a womb-like state and be ‘born again’ is vetoed by others as being simply too dangerous. Barnes’ health was at times extremely perilous. Berke writes, “Some residents insisted that she be sent to mental hospital as soon as possible,” in defiance of Laing's vision of replacing conventional medical options. Barnes became very ill while in the Hall: a uterine hemorrhage required hospital treatment. Back at the Hall, Laing says that “She looked as though she was approaching a state very near actual death” (1972, 18).

Barnes’ deleterious mental and physical state, it seems, afforded her a special status in the Hall. No wonder: here was the pioneer of madness; she was enacting a version of Laing’s psychosis-as-journey template. The resident and helper Noel Cobb, in his account of life at the Hall, mentions a woman who was enraged at the ‘special treatment’ Barnes was receiving and who refused to allow Cobb to take food to Barnes (Cobb MS Laing L169/1-3). The household, apparently, came close to breaking point with the extremity of Barnes’ condition placing the Kingsley Hall experiment in danger at times. What caused the greatest problem for Mary is what led to her acclaim outside the house: her paintings. Others thought she was trying to take over the household by putting up her work everywhere, and she was told to remove her paintings. It was only thanks to what Berke presents as his “heavy politicking on Mary’s behalf” that she was allowed to remain in the community. It is not clear if the problem here was also related to Barnes’ non-payment of rent. In 1968, she tells us, she was given notice to leave the community several times because of non-payment (1991, 229, 167, 261, 292). *Two Accounts,* then, presents a household beset by quite ordinary problems, despite its ostensibly experimental and radical focus, and despite the dangerousness of Barnes’ plight. How do the bills get paid? Who is in charge? How far can one person be allowed to pursue their course if it troubles others? As “a place to be” the Hall was far from Laing’s poetic fantasy.

The sociologist Nick Crossley has described Kingsley Hall as a ‘working utopia’, a sort of lab in which radical anti-psychiatric ideas could be tested out (1999, 809-830). Laing himself presents the Hall as an experiment in his “Metanoia” essay. Archival sources also present this outlook. For two Hall residents, both of whom shared at times in the care of Barnes, it was “a noteworthy exploration of madness” (Noel Cobb MS Laing L169/2) and “an experimental residential community” (Leon Redler MS Laing L221/15A). Berke underlines this view when he writes of Laing wanting to establish a place where a small number of people might safely ungergo “a psychosis “trip” … In this way they hoped to learn about the entirely of the psychotic experience.” Living in the Hall, those from a professional therapeutic background might pursue their experiment as informal participant-observers. Within these terms, Berke sees Kingsley Hall as a success *for him*: “I have received expert teaching on how and why a person can manage to tie her life into knots and then forget where the last strand begins,” he writes of his learning experience (1991, 83, 88). There is no sense in Barnes’ narrative, however, that the community represented simply an experiment for her. Rather, after periods of hospitalization and poor mental health, the implication is that Kingsley Hall was her last chance. Not everyone, it seems, was in the same utopian 1960s boat. At the same time, however, Barnes is more than simply an object of research, and more, even, than a focus of research about whom Berke and others at the Hall care about deeply. In writing her account of her life at Kingsley Hall, she takes up a position as a researcher in an important experiment. *Two Accounts’* dual narrative form enables her to authorize her own version of her life story and life at the Hall and to become, with Berke, a co-producer of research, a reflexive researcher in her own right.

While the Hall could be a problematic environment, it is nevertheless presented as being far more positive than the ‘enlightened’ psychiatric hospital. Laing implies the inadequacy of the therapeutic community, but Berke is explicit on this point. While “people like Max [Maxwell Jones, with whom Berke worked briefly at the Dingelton Hospital in Scotland] felt sure enough of themselves to allow the staff and patients a large measure of self-determination,” Berke says, it was far more common for such “communities,” with their pretensions to patient self-expression and autonomy, to become “organised brainwashing” with conformity enforced by nurses and administrators. Berke adds testimonial authority by giving an account from his time as a medical student in the USA when he witnessed the unnecessary containment by “the community” of a patient who had only been ecstatically dancing (1991, 90-93). The implication is that such an injustice could never occur at Kingsley Hall. Significantly, however, neither Berke nor Barnes discuss the event that the US author, former Kingsley Hall resident and the initial chair of the Philadelphia Association, Clancy Sigal, has given in his fictionalized account of his own experience, *Zone of the Interior* (1976), and has discussed directly elsewhere (2005). By his own account, Sigal had a mental breakdown *and* breakthrough in Kingsley Hall, but part of this involved the decision to leave the community. After doing so, he was visited and physically held down by Laing, Berke and others who thought Sigal suicidal. They injected him with a tranquilizer and returned him to the Hall. He had to pretend to be a ‘good’ resident in order to finally get out^6^. Writing in 2007, Berke admitted that he and Laing had treated Sigal appallingly. What must have been a major event in the life of the community, and one that could be understood as giving the lie to visions of anti-psychiatric harmony, is given no place in the text of *Two Accounts.* It is barred from the ‘official’ version of the Hall.

Such an event is far from Laing’s ‘template’ of madness as a voyage or the ecstasy he imagined in his ‘place to be.’ But there was certainly a place for the ecstasy of dancing at Kingsley Hall, especially after dinner, and for Barnes it was therapeutic. “What helped me to get a feel of my own body was dancing,” she explains. Her therapy, though, becomes increasingly conventional, more congruent with psychodynamic tradition. While she is initially cared for by Berke, who spends whole days with her at Kingsley Hall, their relationship changes. As Barnes improves, Berke notes that “meetings also became more structured. They took place at a set time and place each week,” and they occurred in Berke’s consulting room outside Kingsley Hall. He understands her progress as having been enabled by her internalization of himself: “her internalised relationship with ‘Joe’ helps her in times of emotional difficulty” (1991, 126, 360). Such internalization was seen as crucial by the psychoanalysts D. W. Winnicott, Ronald Fairbairn and Harry Guntrip; and in presenting himself as Barnes’ ‘good object’ and key to therapeutic change, Berke aligns himself with object relations-orientated psychoanalysis.

## Berke’s psychoanalysis and Barnes’ spiritual quest

Psychoanalysis has only a minor presence in Barnes’ narrative. She mentions a correspondence with Anna Freud that she had hoped would lead to therapy, but Anna wrote saying that Barnes would be unsuitable for analysis. In hospital, though, Barnes was visited by a psychoanalyst who, while saying little, says Barnes, “touched something inside” and inspired her to be a helpful, compliant patient who could leave hospital and take up nursing again. For Berke, psychoanalysis is very important, and here he differs not only from Barnes but also from Laing in *The Politics of Experience* and his writing on the Hall. Barnes’ regression was for Berke “the means by which Mary sought to deny jealous anger and all the guilt attendant on it … ‘going down’ [was for Mary] in the service of avoiding painful feelings [and] was one of Mary’s most vital intra- and interpersonal defensive manoeuvres.” Barnes’ regression is not a romantic ‘going back’, part of a journey: it is, for Berke, a regression related to her desire to have babies. “Mary set out to make babies by turning herself into a baby,” he explains (1991, 54-55, 63, 367).

Berke also fundamentally, and very psychoanalytically, relates Barnes’ madness to sexuality (a link absent from from Laing’s thinking): “Mary continually twisted her strong sexual drive up, over and into itself… Death was the only way out, first the death of the hated others whom she mistakenly blamed for her predicament, and if this failed, then the death of herself— death in order to escape from sexual feelings.” Berke relates Barnes’ It, ‘her rage when she felt threatened by feelings which were too hot to handle’, to Freud’s id. “Clearly,” writes Berke, “Mary referred to the same sexual energies and drive when she used the word [it], but to energies which had been transformed by the guilt from the sphere of creation to that of destruction.” For Berke, ‘Identification’ is not an outdated term to be sneered at, as Laing does, but a term with significant explanatory power. According to Berke, ‘It’ was, “the psychic trick Mary played on herself in order to achieve her aim of getting back inside another’s body” (1991, 54, 62-3, 367-9).

Just as Berke differs from Laing in respect of psychoanalysis, so he does in relation to mysticism. Berke’s final chapter, “Untangling Mary’s Knot,” alludes to Laing but in a rather non-Laingian way. For the mystical Laing in his 1970 verse volume *Knots,* problems of tangled relationships, minds and bodies cannot simply be ‘untangled’. Rather, they are Gordian knots that must be ‘cut through’ in a moment of *sartori,* or sudden illumination that transcends illusory problems.^7^ But Berke is not at all mystical in orientation in *Two Accounts*, nor is he in accord with Barnes. “Mary sees her ‘going down’ and ‘return’ as a spiritual journey rather than a sexual struggle,” he remarks. This is a fundamental difference in views and something we might expect him to discuss further, but he does not. He continues, saying of the two viewpoints that they “are not incompatible. My emphasizing the mortifications of the flesh does not detract from the realization of the soul. Most Christian mystics would agree with me” (1991, 370). Three brief sentences: an acknowledgement of differences that borders on a glossing over of them. Yet, we must note, the incongruity in views, foregrounded by the dual narrative form, does not simply detract from the coherence of the text or undermine its argument—the value of withdrawal into madness. Rather the differences here present Barnes’ achievement of difference from others.

Barnes understands her experience very much in spiritual terms. “Being received into the (Catholic) church, into the mystical body of Christ, was the most important event in my life,” she says. Prior to Kingsley Hall, Barnes had wanted to become a nun and might have been one “if God had not rescued [her] through mental breakdown.” Her faith is evident most clearly in her religious-themed painting and reflections upon them. Writing of her *Temptations of Christ*, for instance, she says, “It’s a painting. It’s finished”—perhaps referencing John 19:30. And then she elevates her subject matter by moving into poetry: alternating lines of accentual, non-syllabic verse (four stressed syllables a line, followed by five, then four, then five stressed syllables):

The Devil fades, Christ is at peace.

The picture seems to look at me. Did I do it?

Yes, it was a movement of me, from me.

Strange. Power. Awe, of God, of myself.

She goes on to write, “Sin to me as a whole was a state of alienation of the self,” and here she is close to the Laing of *Politics*. For both, moving from alienation towards authenticity is to move from fragmentation towards wholeness, and wholeness equals holiness. Therapy and religion are thoroughly inter-implicated. Barnes looks forward, in the end, to moving beyond illusory egoic consciousness into a state of all-encompassing heavenly union. “May all be shattered into God, split into the light of heaven,” she writes (1991, 50, 219, 152, 75, 17). In her focus on spirituality, then, there is a correspondence with Laing, but, as Showalter notes, Barnes’ experience is far from the “epic, heroic and masculine, a psychic pilgrimage” (1991, 232) of Laing’s *Politics*. Barnes is not a cosmonaut of the vast unknown psychic interior, an inner spacewoman; nor, I would add, is she on a mission of ideological rebellion to open up new ground beyond the civilizations of East and West. The text presents her, rather, as a desperate woman engaged in a project to find livable ways of existing with her own demons—especially what she terms “It,” her anger—and with others. In this task she is not merely sustained by her faith. Her faith deepens and renews her commitment to therapy, just as therapy renews and deepens her faith. Kingsley Hall, it is implied, provides a means for her to divest herself of the alienating effects of socialization and to key into her core self, which in turn is connected to God.

What kind of parallels might be made between Barnes’ life and work and Christianity more widely in the 1960s? Barnes was very much a Catholic, and when we think of countercultural religion, it is likely that we will think first of Protestant new religious movements, such as the Jesus People. It is difficult to relate Mary Barnes’ Catholicism to contemporaneous Catholic movements or currents; she does not represent a current or particular style of ‘countercultural’ Catholicism. Of course, Catholics, and sometimes prominent ones, such as Thomas Merton and Daniel Berrigan, participated in or lent support to civil rights and anti-war activity in the 1960s, and in this respect we might speak of countercultural Catholicism. The influential Jesuit John Courtney Murray, who contributed to Vatican II, even experimented with LSD (but certainly did not advocate that young Catholics ‘turn on’ to psychedelics). The Catholic 1960s can be understood most broadly in terms of the second Vatican council (1962-65), which brought about changes, especially the transformation of liturgical practice that might be considered the church’s own 1960s revolution.

There is one Catholic movement, The Catholic Workers, founded in the US in the 1930s and practising a kind of Christian anarchism, that can be compared in some respects to Kingsley Hall. The CW website states: “It is unlikely that any religious community was ever less structured than the Catholic Workers […] There is no board of directors, no sponsor, no system of governance, no endowment, no pay checks, no pension plans. Since Dorothy Day's [the founder’s] death, there has been no central leader.” The disdaining of hierarchy and the celebration of the communal that characterises the Catholic Workers certainly meshes with the counterculture, as does their active concern with social justice. There is no evidence, though, of Barnes or anyone at the Hall having had any contact with the Catholic Workers. It is best to think of Barnes as practising a unique version of countercultural Catholicism. While rooted in an experimental 1960s community and often framed by herself in terms of a movement towards a true self, Barnes’ spirituality can be compared to that of a Christian mystic. She does not write of mystical visions but gestures towards mystical oneness with the divine in expressing her desire that “all be shattered into God” (1991, 17). Like many mystics, she underwent great privations (such largely eschewing food for extended periods) that could be understood as a means of purifying the soul. Also, her withdrawal from the world (albeit a supported from of withdrawal) and its corrupting of the self has affinities with the mystic’s removal of him- or herself from the world in order to know God better. We might think, too, of Barnes’ painting, particularly the painting of Christ’s passion, as a form of meditation. Her Christianity is crucial to her recovery.

She does, we are told, find satisfying ways of living. She sets out on a journey to ‘find’ an authentic self and ‘finds’ one. *Two Accounts* fits well with what the medical sociologist Arthur Frank calls, in his typology of illness narratives, a quest narrative. “Quest stories,” Frank writes, “meet suffering head on; they accept illness and seek to *use* it. Illness is the occasion of a journey that becomes a quest” (1995, 115). Fundamentally, and here quite congruent with Laing, Barnes’ withdrawal and regression is understood by *Two Accounts’* narrators as necessary, as her path towards a better life. In this sense, her emotional difficulties have a ‘use’, but it would be more accurate to say that her difficulties have a *truth* value: they are presented as being more truthful than her more socially adaptive behavior as a nurse. Her narrative, in Adame and Hornstein’s terms, is not characterized by a traumatic interruption after which she returns to a prior state of affairs. Nor is Barnes’ story fundamentally one that presents alternating periods of emotional stability and instability. At root, the story is one of “Revelation/Purposeful Suffering,” a story in which emotional crisis “changes the person’s outlook on life, concept of themselves, and direction of post-distress narrative” (Adame and Hornstein 2006, 144). The quest narrative has strong Christian connotations (Woods 2014, 121). Barnes, who writes a story very much as a Christian—and hers is a story of great pain, redemption and rebirth—brings out the Christian associations of the quest-based illness narrative.

## *Two Accounts* and after

In his Foreword to *Two Accounts’* 1991 edition, Berke is even more psychoanalytic and critical of Laing. “Ronnie erred in thinking that most individuals, especially those diagnosed as schizophrenic, could go through this process of self-renewal,” he writes, and draws upon the ideas of the Hungarian-born psychoanalyst Michael Balint (1968) in pointing out that regression might be malign (irreversible, negative) or benign (temporary, beneficial). “Mary’s regression was both benign and malign,” he says. It was benign enough, however, for her (with Berke’s support) to regress fruitfully and recover. Such was not the case, though, for others at the Hall. Looking back, Berke reflects that “Most [residents] remained stuck in a variety of self-annihilating states” (1991, 7). Barnes, then, had her “sheet anchor,” the reliable means of support that Jesse Watkins speaks of Laing’s *Politics of Experience*. But not every one enjoyed such support. If the purpose of *Two Accounts* was to present the Hall as a success, as a model for the treatment of psychosis, it seems that Barnes’ story can only indicate qualified success. According to Berke, and here we might compare his presentation of isolated suffering with the images of desolation found in Cobb’s account of life at the Hall (MS Laing L169/1-3), the Hall cannot simply be taken as a model.

Berke left the Philadelphia Association in 1970 and with Morton Schatzman set up his own psychotherapeutic organization, the Arbours Association. In his 1991 epilogue *to Two Accounts*, Berke states, “For a long while I had observed that an unstructured community like Kingsley Hall was unsuited for helping people in acute emotional distress. The support was not as immediate nor as intensive as it needed to be” (377). He and his colleagues founded a Crisis Centre in Crouch End, North London, and a household nearby for less severely disturbed people. These households were an attempt to provide the ‘structure’ he found lacking in Kingsley Hall. It seems that post-Kingsley Hall, Berke found Esterson’s argument that a therapeutic community cannot be a ‘free for all’ more persuasive than Laing’s rather laissez-faire attitude. We can see, too, a movement away from the optimistic 1960s view that appropriate structures will arise organically and so need not be set out in clear frameworks.

Berke’s split from Laing is played out in Barnes' writing. In her unpublished sequel, *Attic-Archway-Devon*, Barnes details her move from Kingsley Hall to Archway, North London (to another Philadelphia Association household) and then to Devon. She is explicit in stating that she was divided between Berke (the Arbours Association) and Laing (the Philadelphia Association). In Chapter 4, she is “furious with Joe (the source of so much light and creativity in me),” but in relation to Laing, “terrified he would be angry with me if I was under Joe, or when I was impelled to leave Archway for Devon I feared Ronnie would ‘slay me’ for getting up, and out, and into Devon” (Barnes MS Laing V38.) What is interesting here, and what speaks of therapeutic advancement, is Barnes’ ability to bear strong and difficult feelings without collapsing into withdrawal or being consumed by the murderousness of “It.” There is evidence of this, too, in her epilogue (entitled “Finale”) to the 2002 Other Press edition of *Two Accounts* in which she admits her difficulty with the Christian injunction to love her neighbor (363). She is now, though, able to find the right words: previously unbearable affect is rendered manageable through symbolization.

As the 1970s progressed, Laing (and Barnes) became increasingly interested in the trauma of birth, in perinatal psychology and in the practice of rebirthing introduced to the UK by the US therapist Elizabeth Feher. Laing’s promotion of rebirthing caused conflict in the Philadelphia Association and was a significant reason for his resignation as chair in 1981 (see Cooper et al. 1989: 28-30). In *Something Sacred*, a volume of Barnes’ interviews with the psychotherapist Ann Scott from the 1980s, Barnes responds to a question about “large group ‘birthing’ events” by saying, “I think I had a good experience of it.” She had, she continues, “already known very early … pre-birth feelings in Kingsley Hall and I consider it a very powerful means of therapy.” She is defensive of Laing, arguing that “whatever Ronnie has said or done in certain circles, he’s still absolutely brilliant … He was the burning light that led the way into the forest”, even if now “the light has gone off on its own track” (Barnes and Scott 1989, 363). Barnes is able to both agree with Laing and mark her distance from him: she can illuminate her own ‘track.’ She emerges very much as her own person.

Barnes’ affinity with Laing is strongest when speaking of the spiritual dimension of regression:

I do think there are similarities between the therapeutic household and some religious practices… I certainly fasted, I had solitude, and I knew physical pain in the body without psychical sickness. All these things various religions have been very aware of in their conscious desire for wholeness and holiness for centuries (1991, 27-8, 79, 56).

Here Barnes echoes Laing in *The Politics of Experience,* a text that, as Gavin Miller has pointed out, can be read as presenting psychosis as “the lost essence of early Christian religious experience” (2009, 15). Regression for Laing, and Barnes, is a form of religious practice, but the spiritual potential of regression goes beyond Christianity to embrace religion more generally for both Laing and the devout Catholic Barnes. The mad person, given the right environment, might express the awareness common to various religions. We are offered a countercultural version of perrenialism.

In *Something Sacred*, Barnes, as well as reaffirming her spirituality, is now able to distance herself from the experience of Kingsley Hall and register how disturbing she was for others: “It’s practically impossible,” she says, “if you’re the one that’s really down and being helped, to understand the extent of the anxiety you’re provoking in your fellow residents.” What helped her to further recognize others’ separateness and to critically reflect on her own past—and, perhaps, move beyond her post-Kingsley Hall divided loyalties—was seeing the late seventies play about her own life, *Mary Barnes* by David Edgar. Watching the play, she says, “I could see how necessary it is to have more of an objective view and not to think that everybody is just like you” (1989, 17-18, 39). Writing, then, once again proves therapeutic—not Barnes’ own this time (as was the case with her anti-psychiatric fairytales, and, implicitly with the writing of *Two Accounts*), but someone else’s words.

## Conclusion

Returning to the words of Barnes and Berke reveals both divisions and affinities between the two of them, and affinities and differences between each of them and Laing. Differences are highly significant, particularly the ways in which Barnes, in and through the dual narrative form, is able to differ from others. Far more than an exemplary patient, victim or dupe, her particular vision is discernible. *Two Accounts*’ ‘journey’ rebuffs Laing’s version—his ‘route map’—of metanoia in which the psychotic ‘voyage’ is analogous to the coming up and coming down of an acid trip, or a psychedelic voyage of just a few days. But we ought not to overlook what Barnes, Berke and Laing have in common. Certainly, *Two Accounts* problematizes Laing’s 1960s utopianism, and Barnes’ and Berke’s narratives and post-*Two Accounts* comments represent different understandings of regression. But they both agree that Barnes’ therapy was a success. Barnes and Berke still represent countercultural hopefulness: Barnes in her focus on spiritual redemption and the reclamation of wholeness and authenticity, and Berke with his ‘hands-on’ psychoanalysis. Implicit in the writing of both is the belief that, in the right circumstances, and for at least some people, it is possible to strip away the excrescences of socialization in order to get in touch with a core self that can form the basis of eventually rearticulated and differently integrated selfhood. This view of the self, understood in terms of surface and depth, might not be nearly as compelling today as it was then, but it is a view that both Barnes and Berke share with Laing.

Kingsley Hall was an experiment of a previous age, of course. As Torn (2012) points out, twenty-first century readers might well be shocked at the relative lack of therapeutic boundaries and the lack of regulation in the household. Yet renewed attention to the Hall testifies to continuing interest in the household (Fowler 2001; Harris 2012; McGeachan 2014). The Hall was the first Philadelphia Association household, and there are two in London today. These are far more structured and regulated than the Hall, but, writing in 2010, Paul Gordon, a therapist at one of the remaining households, wrote that the story of Barnes “continues to bring some people to PA houses today” (11).^8^ Kingsley Hall is now a community centre. One group, The Friends of East End Loonies (F.E.E.L), which sympathizes with the radical intent of the Barnes-Berke era Hall, holds discussions about mental health in the Hall.

The Kingsley Hall experiment may not provide a straightforward alternative to biomedical conceptions of mental illness and its treatment, but analysis of *Two Accounts* might, I suggest, provide useful starting points for those interested in social psychiatry today, as well as to practitioners and scholars interested in mental health, spirituality, and metaphors of therapeutic progress. The reading of the text offered here will, I hope, inform further re-evaluation of Laing and his circle, and provide a useful reference point for readings of as yet unconsidered accounts of life at Kingsley Hall in the R. D. Laing archive at the University of Glasgow.
